# The interaction between GCN2 and eIF2 mediates the resistance of cotton bollworm to the *Bacillus thuringiensis* Cry1Ac toxin

**DOI:** 10.1371/journal.ppat.1013510

**Published:** 2025-09-15

**Authors:** Caihong Zhang, Jizhen Wei, Ningning Li, Zaw Lin Naing, Ei Thinzar Soe, Jinrong Tang, Huan Yu, Fengyun Fang, Gemei Liang

**Affiliations:** 1 State Key Laboratory for Biology of Plant Diseases and Insect Pests, Institute of Plant Protection, Chinese Academy of Agricultural Sciences, Beijing, PR China; 2 State Key Laboratory of High-Efficiency Production of Wheat-Maize Double Cropping/College of Plant Protection, Henan Agricultural University, Zhengzhou, China; INEM: Institut Necker-Enfants Malades, FRANCE

## Abstract

Deciphering the molecular mechanisms underlying insect resistance to Cry toxins produced by the soil bacterium *Bacillus thuringiensis* (Bt) is crucial for the sustainable utilization of Bt-based products. Previously, we identified that the reduced expression of the *CAD*, *ABCC2* and *ABCC3* genes through a eukaryotic translation initiation factor 2 (eIF2) is associated with Bt Cry1Ac resistance in the cotton bollworm, *Helicoverpa armigera*. Here, we found that an eIF2 alpha kinase, GCN2, is highly conserved in Lepidoptera insects. Its inhibitor GCN2iB (ATP-competitive inhibitor of serine/threonine protein kinase, stress-responsive kinase) could decrease the toxicity of Cry1Ac, and the GCN2 enzyme activities decreased after larvae fed Cry1Ac. The expression level of the *HaGCN2* gene and the enzymatic activity of its corresponding protein were significantly down-regulated in the BtR resistant strain. Moreover, both BiFC and Y2H assays demonstrated that eIF2 could interact with GCN2. Finally, the silencing of *HaGCN2* expression not only decreased the protein and phosphorylation levels of eIF2α but also reduced the expression of *HaCAD*, *HaABCC2*, and *HaABCC3*. This consequently led to a decrease in the toxicity of Cry1Ac toward *Helicoverpa armigera*. These results indicate that GCN2 conferring Cry1Ac resistance in *H. armigera* through regulating the expression of eIF2. This finding deepens our understanding of the transcriptional regulation of midgut Cry receptor genes and the molecular basis of insect resistance to Bt Cry toxins.

## Author summary

Deciphering the molecular mechanisms of insect resistance to Bt Cry toxins is critical for sustainable Bt product use. Previously, we found reduced expressions of CAD, ABCC2, and ABCC3 via eIF2 correlated with Cry1Ac resistance in *Helicoverpa armigera*. Here, we show that GCN2, an eIF2 alpha kinase, is conserved in Lepidoptera insects. Its inhibitor, GCN2iB, decreases Cry1Ac toxicity, and GCN2 activity declines after larvae eat Cry1Ac. In the Bt-resistant strain, HaGCN2 expression and enzyme activity are significantly downregulated. Both BiFC and Y2H assays confirm interaction between eIF2 and GCN2. Silencing GCN2 reduces eIF2α protein and phosphorylation levels, as well as HaCAD, HaABCC2, and HaABCC3 expression, decreasing Cry1Ac toxicity toward *H. armigera*. These results suggest GCN2 regulates Cry1Ac resistance by modulating eIF2 expression, advancing our understanding of midgut receptor gene regulation and insect resistance to Bt toxins.

## Introduction

The Gram-positive entomopathogen *Bacillus thuringiensis* (Bt) is the most widely used biopesticide worldwide due to its highly specific activity and environmental safety [1]. Bt biopesticides and genetically modified Bt crops have been developed and widely used for pest control [2–4]. However, high adoption of Bt crops and concurrent use of Bt pesticides represent high selection pressure for insect resistance evolution. To date, cases of field-evolved resistance to Bt products have been reported in at least eleven pest species [5]. The economic and environmental significance of Bt insecticides highlights the importance of clarifying the molecular mechanisms by which insects develop resistance to Bt.

As currently understood, the mode of action of Bt Cry toxins involves multiple steps that occur in the larval midgut, and the binding of Bt Cry toxins to functional receptors in the midgut is a key step in this complex toxicity process [6–8]. The identified midgut receptors of Bt Cry toxins include cadherin (CAD) and ATP-binding cassette (ABC) transporter proteins [9,10]. The downregulation of midgut Cry receptor genes is one of the principal drivers of Bt resistance evolution in diverse insects [11–13], and mutation of these receptors can lead to resistance [14–16]. Recently, the transcriptional factors forkhead box protein A (FOXA) and GATA modulated the transcription of Cry1Ac receptor genes in insects [17–19]. Further, MAPK-activated PxJun and (fushi tarazu factor 1) FTZ-F1 suppress the expression of Bt Cry1Ac receptors to confer Cry1Ac resistance [20,21].

Lately, we also found that a eukaryotic translation initiation factor 2 (eIF2) resulted in the downregulation of HaCAD, HaABCC2 and HaABCC3 receptor genes [22]. General control nonderepressible 2 (GCN2), as a kind of eIF2 alpha protein kinase has been shown to control eIF2 alpha phosphorylation in response to diverse stresses [23–25]. Active GCN2 phosphorylates the α-subunit of the eIF2, this phosphorylation event leads to the inhibition of general translation initiation. At the same time, it activates the translational derepression of mRNAs, thereby regulating the cellular responses to certain stress conditions [26–28]. However, it remains unclear whether eIF2 is regulated by GCN2 to participate in the response to Cry1Ac toxicity and resistance.

In this study, we further probed the mechanism of Bt Cry toxin resistance in *H. armigera*. We successfully cloned and characterized the *GCN2* gene of *H. armigera*. GCN2 inhibitor GCN2iB decreased larval susceptibility to Cry1Ac toxin in the susceptible strain. *GCN2* expression was significantly lower in Cry1Ac-resistant strains than in the Cry1Ac-susceptible strain. More importantly, GCN2 interacts with eIF2, and silencing of GCN2 expression downregulated eIF2 phosphorylation and eIF2 expression, finally reduced receptors transcription. We uncovered that the eIF2 is modulated by the GCN2 kinase signaling pathway. Our results provide important insights into the transcriptional regulation of Cry1Ac receptors, which in turn enhance our understanding of the evolution of insect resistance.

## Results

### GCN2 sequence and phylogenetic analysis

The full open reading frame (ORF) (GenBank Accession no. PQ002176) of *HaGCN2* is composed of 4695 nucleotides, encoding 1565 amino acid residues. The predicted molecular mass of the HaGCN2 protein is 175.03 kDa, with a predicted isoelectric point of 6.28. The HaGCN2 protein has four predicted conserved domains showed in S1 Fig, RWD domain of eIF-2-alpha kinase GCN2 and related proteins (RWD–GCN2). Protein Kinases, catalytic domain (PKC–like super family). Catalytic domain, repeat 2, of the Serine/Threonine kinase, eukaryotic translation Initiation Factor 2-Alpha Kinase 4 or General Control Non-derepressible-2 (STKC–eIF2AK4–GCN2–rpt2). Class II tRNA amino-acyl synthetase-like catalytic core domain (Class–II–aaRS-like-core super family).

**Fig 1:**
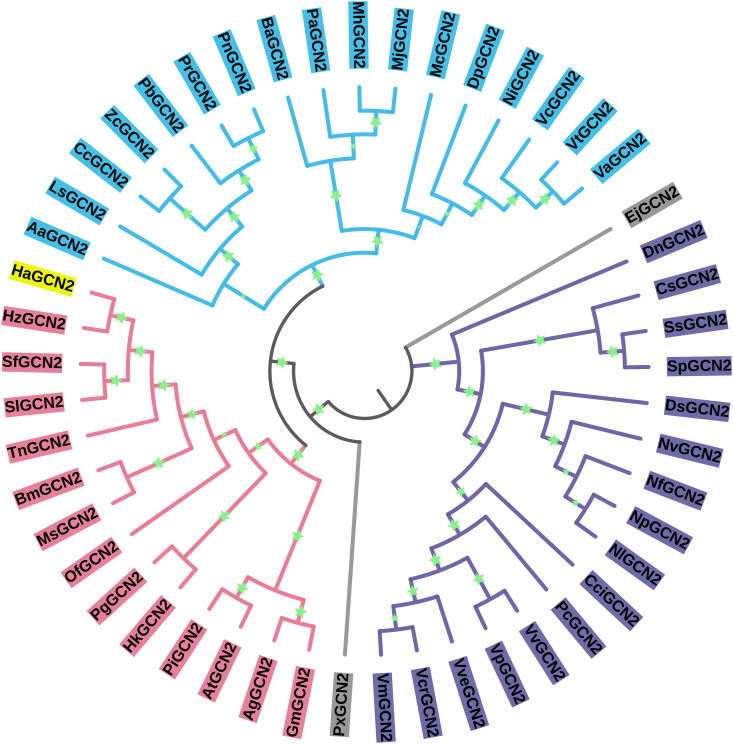
Phylogenetic tree of GCN2 genes from insects. Phylogenetic trees were constructed using MEGA 5.0 software employing the “p-distance” model with 1000 bootstrap replicates and the Neighbor-Joining (NJ) method. Full-length amino acid sequences of GCN2 genes were retrieved from GenBank database at 49 aligned amino acid positions (S2 Table). The green stars indicate the bootstraps/metadata of the values.

A model-based phylogenetic analysis demonstrates that GCN2 proteins from different insect orders are clusted in independent branches and are evolutionarily conserved (Fig 1), the phylogenetic tree revealed close relationship among GCN2 proteins from Lepidoptera and HaGCN2, which indicated that these GCN2 proteins are homologous.

### Tissue expression profiles of the *GCN2* gene in 96s and BtR strains

**Fig 2:**
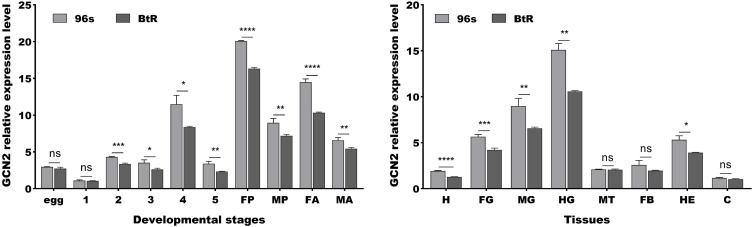
Relative expression level of GCN2 in the resistant and susceptible strains of *H. armigera.* A Relative expression levels of GCN2 in egg, first -fifth instar larvae (1-5), female pupae (FP), male pupae (MP), female adults (FA), male adults (MA) of the 96s and BtR strains. B Relative expression levels of GCN2 in head (H), foregut (FG), midgut (MG), hindgut (HG), malpighian tube (MT), peritrophic membrane (PM), fat body (FB), hemolymph (HE), and cuticle of 96s and BtR strains (C). Asterisks indicate significant differences in three treatments between the 96s and BtR strains (ns, not significant, * *P* < 0.05, ** *P* < 0.01, *** *P* < 0.001, **** *P* < 0.0001, *t*-test).

Expression analysis of the *GCN2* gene by PCR at various developmental stages in the 96s and BtR strains indicated that the expression level was higher in the second-fifth instars, female pupae, male pupae, female adults, and male adults of the 96s strain (*P* < 0.05) (Fig 2A). Moreover, expression analysis of *GCN2* in the different tissues of fourth-instar larvae in the 96s and BtR strains showed that it was significantly highly expressed in the head, foregut, midgut, hindgut and hemolymph tissues of the 96s strain, this was in contrast to its expression in the malpighian tube, fat body and cuticle (*P* < 0.05) (Fig 2B).

### GCN2 inhibitor reduced the toxicity of Cry1Ac to *H. armigera*

**Fig 3:**
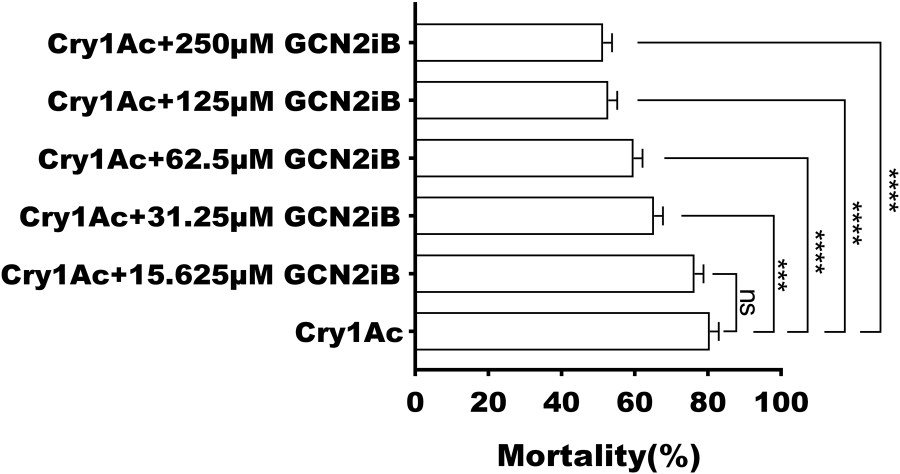
Effects of GCN2 inhibitor GCN2iB on larval mortality of Cry1Ac-treated *H. armigera.* The mortality of susceptible strain larvae after feeding on Cry1Ac (8 µg/g, which resulted in approximately 80-90% larval mortality based on previous results) and GCN2iB (15.625, 31.25, 62.5, 125 or 250 µM). Each error bar represents the standard error of the mean from three biological replicates. Asterisk shows significant differences between Cry1Ac and the different treatments (ns, not significant, *** *P* < 0.001, **** *P* < 0.0001, *t*-tes*t*).

**Fig 4:**
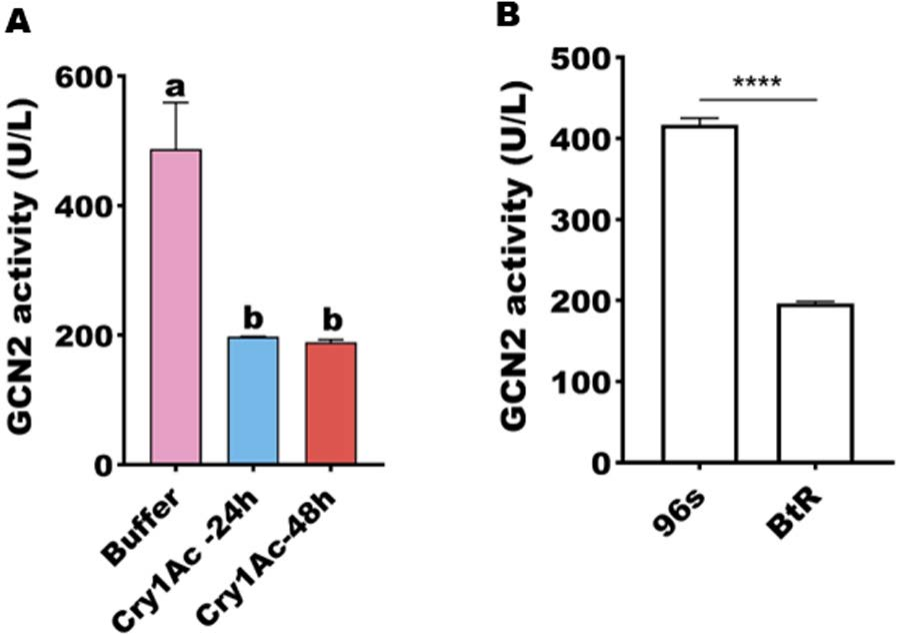
The enzyme activity of GCN2 in *H. armigera.* A GCN2 activity after treatment of larvae with Cry1Ac protoxin. Buffer (50 mM Na_2_CO_3_- NaHCO_3_, pH 10, without treatment) used as control. B The enzyme activity of GCN2 between susceptible strain and resistant strains (*P* < 0.0001). Each error bar represents the standard error of the mean from three biological replicates. Different letters also indicate significant differences among three treatments (*P* < 0.05, Tukey’s test). Asterisk shows significant differences between the 96s and BtR strain (**** *P* < 0.0001, *t*-test).

Since GCN2 is known to phosphorylate eIF2 in humans, we first wanted to see whether its homolog in *H. armigera* might have a similar function. Initially we tested the effect of the specific inhibitor (GCN2iB) on the toxicity of Cry1Ac. The presence of the inhibitor resulted in a decrease in susceptibility (Fig 3), And the activity of GCN2 was found to have decreased 24 and 48 h after feeding with sublethal dose of Cry1Ac (Fig 4A). Furthermore, the enzyme activity of GCN2 was lower in the resistant strain than the susceptible strain (Fig 4B).

### GCN2 interacts with the eIF2

**Fig 5:**
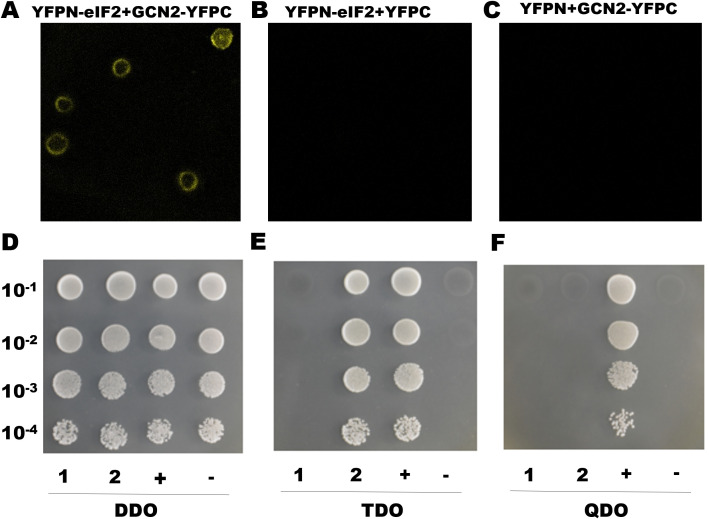
The interaction between eIF2 and GCN2. A-C BiFC analysis of interactions between eIF2 and GCN2 proteins. The Sf9 cells were co-transfected with the indicated plasmid pairs: YFPN-eIF2 + GCN2-YFPC, YFPN-eIF2 + YFPC, YFPN + GCN2-YFPC). The images were captured 24 h post-transfection using confocal microscopy. YFP fluorescence represents protein-protein interactions. D-F Yeast two-hybrid analysis of the interaction between eIF2 (Bait) and GCN2 (Prey). Gradient concentrations of the yeast cells co-transformed with: (1) self-activation verification: PGBKT7-eIF2 + PGADT7, cells grew on DDO plates but not on TDO or QDO plates, indicating that eIF2 did not exhibit auto-activation. (2) PGBKT7-eIF2 + PGADT7-GCN2 to DDO, TDO and QDO plates. The cells grew on DDO and TDO plates but not on QDO plates, confirming the validity of protein-protein interaction detection. pGBKT7-53 + pGADT7-T and pGBKT7-Lam + pGADT7-T are used as positive (+) and negative (-) controls, respectively.

In BiFC assays, we performed by transiting co-expressing YFPN-eIF2 and GCN2-YFPC or YFPN-eIF2 and YFPC or YFPN and GCN2-YFPC, the peptides YFPC and YPFN were combined to excite fluorescence only when YFPN-eIF2 and GCN2-YFPC combined, otherwise there was no fluorescence excitation (Fig 5A–5C). To further validate whether GCN2 could interact with eIF2, these proteins were screened by the Y2H experiment, we found that the bait eIF2 plasmids (PGBKT7-e2 + PGADT7) could grow well on DDO plate but not grow on TDO and QDO plates, which showed that the transformed Y2HGold yeast strain did not exhibit auto-activation of the eIF2, and eIF2 had no toxic effect on Y2HGold yeast cells (Fig 5D–5F). The cells co-expressing both PGBKT7-e2 and PGADT7-GCN2 successfully grew on DDO and TDO plates but not on the QDO plate (Fig 5D–5F), which indicated that eIF2 could interact with GCN2.

### GCN2

#### RNAi-mediated the eIF2 phosphorylation and expression affected the midgut receptors’ expression.

**Fig 6:**
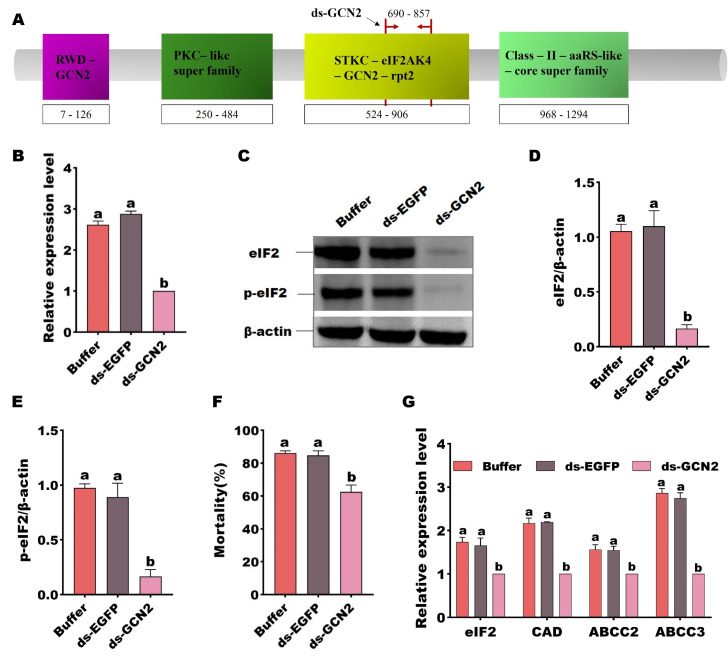
The involvement of GCN2 in eIF2 phosphorylation and *HaCAD, HaABCC2 and HaABCC3* expression. A The ds-GCN2 (690-857AA) sequence is located in the conserved STKC–eIF2AK4–GCN2–rpt2 domain (524-906AA) of GCN2. B siRNA-mediated silencing of GCN2 was detected at transcriptional level in the susceptible strain (*P* < 0.0001). C Protein expression and phosphorylation levels of eIF2 in the larval midgut of the susceptible strain following treatment with ds-GCN2. Phosphorylated eIF2 protein was detected by anti-phosphoserine antibody and non-phosphorylated eIF2 protein was detected by anti-eIF2. Quantified by densitometry and normalized to β-actin. D Quantified eIF2 protein expression level (*P* = 0.001) and E Quantified phospho-intensity following ds-GCN2 treatment (*P* = 0.004). F Effect of silencing GCN2 expression on susceptibility to Cry1Ac protoxin (*P* < 0.0001). G Relative expression of eIF2, *HaCAD*, *HaABCC2* and *HaABCC3* at 48 h postinjection with buffer, ds-EGFP or ds-GCN2. Each error bar represents the standard error of the mean from three biological replicates. Different letters indicate significant differences among three treatments (*P* < 0.05, Tukey’s test).

The *GCN2* gene expression was silenced by microinjection of *H. armigera* susceptible larvae with GCN2 dsRNA (located in the conserved STKC–eIF2AK4–GCN2–rpt2 domain) (Fig 6A) to determine the potential role of the *GCN2* gene in Cry1Ac resistance. The expression levels were reduced after 48 h post dsRNA injection, in contrast, controls treated with buffer or ds-EGFP, did not show any silencing effect on *GCN2* expression (Fig 6B). Assessment of protein levels confirmed that RNAi-induced silencing of *GCN2* was decrease the levels of both total and phosphorylated eIF2 (Fig 6C–6E). The subsequent bioassays revealed that silencing of *GCN2* gene reduced larval susceptibility to Cry1Ac protoxin after 48 h post-injection compared to control larvae injected with buffer or ds-EGFP (Fig 6F), reduce the gene expression of the eIF2 and three receptor genes (Fig 6G). These data are all consistent with GCN2 being involved in the eIF2-mediated pathway modulating Cry1Ac susceptibility via receptor expression.

## Discussion

**Fig 7:**
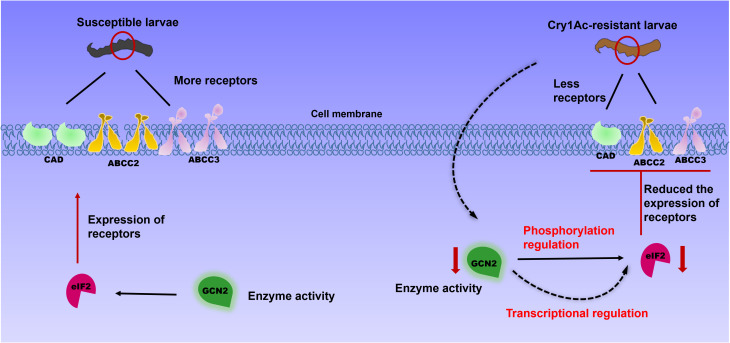
Model of GCN2 regulating eIF2 with enhanced resistance of *H. armigera* to Bt Cry1Ac toxin. Cry1Ac protoxin induced a reduction in GCN2 activity, which resulted in an elevation of the expression level of eIF2. This, in turn, led to the downregulation of receptor gene (CAD, ABCC2 and ABCC3) expression and conferred resistance to the Cry1Ac toxin.

Downregulation of specific Bt Cry toxin receptor genes, such as those encoding cadherin and ABC transporters, in the midgut epithelial cells of insects usually results in high-level Bt resistance in insects. Such a reduction in gene expression disrupts the normal binding processes of the Bt Cry toxins, thereby enabling the insects to withstand the toxic effects of these biopesticides [12,13,29–31]. Some studies have shown that a diverse array of transcriptional factors play crucial roles in participating in the regulation of the expression of midgut receptor genes in insects [17,18,21]. Our latest research has revealed that the eIF2 can regulate the expression of midgut receptors HaCAD, HaABCC2 and HaABCC3 in *H. armigera* [22]. Here, we have shown that GCN2 regulates both of the expression of eIF2 and the phosphorylated form of eIF2 (Fig 6), which results in decreased expression of the HaCAD, HaABCC2, and HaABCC3 genes. This mechanism thereby mediates Bt Cry1Ac resistance in *H. armigera* (Fig 7). These results contribute to understanding different resistance mechanisms and provide guidance for the application of Bt and the management of resistance.

The eIF2 can always be phosphorylated by eIF2α kinases [32]. It has been well-documented that there exist four eIF2α kinases: the hemin-regulated inhibitor (HRI), the double-stranded RNA (dsRNA)-dependent kinase (PKR), the GCN2 protein kinase and the ER-resident kinase (PERK) [33]. Among these, GCN2 as one of the most highly evolutionarily conserved members of the eIF2α kinase family, indicating its fundamental importance across different species in regulating the eIF2α phosphorylation pathway [34,35]. Unlike vertebrates which possess all four family members, insects have only GCN2 and PERK [33]. Here, we demonstrated that the GCN2 inhibitor GCN2iB reduced the sensitivity of *H. armigera* to Cry1Ac (Fig 3), indicating that GCN2 modulates the toxicity of Cry1Ac towards *H. armigera*. Moreover, the reduced GCN2 activities observed in BtR insects (Fig 2), as well as in those treated with Cry1Ac or RNAi (Fig 3), may also contribute to the decreased phosphorylation of eIF2, due to a direct interaction between GCN2 and eIF2 (Fig 5), ultimately leading to increased tolerance of *H. armigera* to Cry1Ac. The regulation of eIF2 by GCN2 is consistent with the result reported in its species that GCN2 is able to be activated and maintain eIF2α phosphorylation levels [25,34,35]. Changes in the expression levels of Bt receptor genes, regulated by alterations in the phosphorylation states of transcription factors and their involvement in Bt resistance, have been reported in several studies [20,21]. For instance, both non-phosphorylated and phosphorylated forms of a MAPK-modulated transcription factor (FTZ-F1), have been shown to regulate the downregulation of Bt Cry1Ac toxin receptors and the upregulation of non-receptor paralogs [21]. These indicated that the phosphorylation modification levels of eIF2 could involve in Cry1Ac resistance. The impact of alterations in eIF2 protein modification levels, regulated by GCN2, on the expression of Cry1Ac receptors will be investigated to elucidate the role of these modifications in modulating Cry1Ac toxicity in future studies.

Importantly, we also observed that the GCN2 gene exhibited markedly high expression levels in the gut tissues of *H. armigera* (Fig 2B). Notably, our findings revealed a significant reduction in the expression of the GCN2 gene in the BtR strain (Fig 2). Corresponding to its enzyme activity decreased by 2.12-fold in the BtR strain (Fig 4B). Also, its enzyme activity of GCN2 were 2.58-fold decrease after 48 h Cry1Ac feeding (Fig 4A). Why does GCN2 respond to effect of Cry1Ac? It has been reported that GCN2 is primarily activated by stress, like nutritional deprivation, viral infection and so on [36,37]. Phylogenetic tree analysis in this study revealed that GCN2 is highly conserved among insect species (Fig 1), suggesting that GCN2 may a functional role under Cry1Ac-induced stress conditions. Importantly, the interaction between GCN2 and eIF2 (Fig 5), together with functional correlation analysis using RNAi (Fig 6), indicates that GCN2 can regulate both the expression and phosphorylation levels of eIF2. And our previous studies have shown the reduced eIF2 gene expression in BtR involved in insect resistance to Cry1Ac [22]. In *drosophila*, FOXO use GCN2 as a sensor to activate stress responses, this allows GCN2 to translate stress signals into the dynamic gene expression programs controlled by FOXO [38]. Further investigation is required to clarify whether GCN2 directly or indirectly regulates the transcription of eIF2. This speculation can be further understood through subsequent studies on the mechanism of GCN2/eIF2 response to Cry1Ac.

Taken together, these results provide new insights into the transcriptional initiation mechanisms of midgut receptor genes and the regulation mechanism responsible for resistance to Cry toxins in this study are critical for sensitive and efficient monitoring and management practices to delay field-evolved insect resistance to Bt products.

## Materials and methods

### Insect strains and cell lines

In this study we used two *H. armigera* strains, a susceptible strain 96s and a Cry1Ac-resistant strain BtR. The 96s strain was initially collected in Xinxiang country, Henan Province, China and has been reared in the laboratory without exposure to any insecticides since 1996. The resistant BtR strain was derived in our laboratory by selection with Cry1Ac (final selection concentration was 360 μg Cry1Ac protoxin per mL of diet) [39] and the larvae present around 3000-fold resistance [16]. All *H. armigera* strains’ raising conditions were as described in Liang et al. [40].

*Spodoptera frugiperda* 9 (Sf9) cells were cultured in the laboratory with Sf-900 II serum-free medium (SFM) (GIBCO, USA), 50 U/mL penicillin and 50 μg/mL streptomycin (Thermo Fisher Scientific) in an incubator 28.4°C [41].

### Toxins and bioassay

Cry1Ac protoxin and activated Cry1Ac were kindly supplied by Insect-Resistant Biotechnology Laboratory, Institute of Plant Protection, Chinese Academy of Agricultural Sciences.

A 24-g diet incorporation bioassay was conducted in 24-well plates, with Cry1Ac protoxin (8 µg/g, which resulted in approximately 80–90% larval mortality based on previous results), control buffer solution (50 mM Na_2_CO_3_- NaHCO_3,_ pH = 10), GCN2 inhibitor GCN2iB (MCE) at concentrations 15.625, 31.25, 62.5, 125 or 250 µM added to the artificial diet [42]. Twenty four first-instar larvae per group and three replicates were tested on each treatment. Larval mortality was recorded after seven days.

### HaGCN2 activity determination

Twenty 4^th^ instar larvae were treated with 24 µg/mL [43] Cry1Ac protoxin to investigate the enzyme activity of HaGCN2, three biological replicates were conducted. After being exposed to Cry1Ac for 24 and 48 h, larval midgut proteins were extracted using 1 mL RIPA lysis buffer (Beyotime Biotechnology) with 10 μL protease inhibitor cocktail (Coolaber), then the midgut tissues were homogenized four times on ice and centrifuged at 4°C and 10,000 g for 5 min, and the supernatant collected. The activity of GCN2 was quantified using insect general regulatory repressor protein kinase 2 (GCN2) enzyme-linked immunosorbent assay kit (mlbio). Briefly, 40 μL of sample and 10 μL sample were mixed, incubated at 37°C for 30 min. After being washed five times, enzyme labeled reagent was added and repeated the above method once, after that separately adding 50 μL chromogenic agent A and B, 37°C avoid light treatment 10 min, the assay can be performed as a stopped reaction using stopping solution. The Microplate Reader (BioTek, Gene Company Limited) was used to monitor the absorbance at 450 after 15 min.

### RNA extraction and cDNA synthesis

The midgut tissues of *H. armigera* were dissected from fourth-instar larvae and homogenized in TRIzol reagent (Invitrogen). Total RNA was extracted following the manufacturer’s protocol and the integrity of RNA was determined via 1% agarose gel. First-strand cDNA for gene cloning was prepared using the HiScript III 1st Strand cDNA Synthesis Kit (+gDNA wiper) (Vazyme), and that for qPCR detection was synthesized using the HiScript III All-in-one RT SuperMix Perfect for qPCR (Vazyme). The synthesized first-strand cDNA samples were stored at -20°C until used.

### Gene cloning

The coding sequence (CDS) of **H. armigera* GCN2* from the GenBank database (*GCN2*, XM_049848510.1). The CDS of *GCN2* were further corrected using our previous transcriptome sequencing data [44]. Gene-specific primers (S1 Table) were designed by Primer3Plus (https://www.primer3plus.com/index.html). The PCR reaction used 2 × Phanta Max Master Mix (Dye Plus) (Vazyme) according to the manufacturer’s protocol. The PCR products were purified (AxyPrep DNA Gel Extraction Kit, Axygen Scientific), subcloned into pEASY-Blunt Simple cloning vectors (TransGen) and then sequenced. The final cloned full-length cDNA sequences of *H. armigera GCN2* genes were deposited in the GenBank database (Accession no. PQ002176).

### Bioinformatic analysis

The amino acid sequences of GCN2 were deduced using the ExPASy translate tool (https://web.expasy.org/translate/). DNA and protein sequence alignments were carried out using DNAMAN 9 (Lynnon BioSoft). Conserved domains were analyzed by the Conserved Domain Database (CDD) at NCBI (https://www.ncbi.nlm.nih.gov/cdd/). Isoelectric point and molecular weight predictions were analyzed at ExPaSy-Compute pI/MW (https://web.expasy.org/compute_pi/). The phylogenetic trees of GCN2 proteins (S2 Table) were constructed using MEGA5.0 software with the neighbor-joining (NJ) method following the p-distance model and 1000 bootstrap replicates. Interactive tree of life (iTOL, https://itol.embl.de/) was used to visualize trees.

### qPCR analysis

The gene transcript levels were determined through real-time quantitative PCR (qPCR) analysis using the specific primers listed in S3 Table. The qPCR experiment was performed in the QuantStudio 6 Real-Time PCR System using Taq Pro Universal SYBR qPCR Master Mix (Vazyme) according to the manufacturer’s instructions as mentioned previously [45]. The relative expression levels of target genes were determined using the comparative CT method (2^−ΔΔCt^) and normalized to the internal control RPS15 (GenBank XM_021326200.1).

### RNA interference

RNA interference (RNAi) of GCN2 was carried out to investigate the regulatory relationships among GCN2, eIF2 and receptor genes and to explore whether the *GCN2* gene is involved in Cry1Ac resistance. Specific dsRNA was designed using the E-RNAi website (https://www.dkfz.de/signaling/e-rnai3/) and synthesized using the T7 Ribomax Express RNAi System (Promega) [46]. The gene-specific primer for the dsRNA templates are listed in S4 Table. An equal volume of buffer (Nuclease-free water) and ds-EGFP were used as negative controls. A total of 1000ng of dsRNA was carried out in newly molted 3^rd^ instar *H. armigera* larvae using a Nanoject III microinjection system (Drummond, USA). Subsequently, to determine the silencing efficiency and receptor genes after injection, midgut tissue was collected from the injected larvae for qPCR analysis. Toxicity bioassay was conducted at 48 h post-injection.

### Protein extraction and western blot

Midgut samples were dissected from fifteen fifth-instar larvae and homogenized several times intermittently on ice in 1 mL RIPA Lysis buffer (Beyotime Biotechnology) with 10 μL Protease inhibitor cocktail (100×), 10 μL Phosphatase inhibitor cocktail (100×) and 10 μL EDTA (100×) (coolaber). And then centrifuged to collect the supernatants. Protein concentration was quantified with the BCA Protein Assay Kit (CWBIO).

Antibodies of eIF2 were generated from synthetic peptides (HUABIO) derived from respective specific amino acid sequences ^318^DEPQQNGASDEDEDC^331^ and ^75^CVDKEKGYIDLSKRR^88^, and other specific antibodies: anti-phosphoserine (abcam, ab9332) and β-actin (LABLEAD, A0101) were commercially purchased. The protein and phosphorylation levels of eIF2 protein was detected by Western blots using β-actin as an internal control in all blots. Midgut proteins were separated on 4–12% SDS-PAGE (GenScript), then transferred to PVDF membranes (Merck Millipore). The membranes were then blocked (5% Difco skimmed milk for protein, 3% Bovine Serum Albumin, BSA, Sigma-Aldrich for phosphorylated protein) at room temperature for 1 h and incubated with the appropriate primary antibody diluted with 3% BSA (0.5% BSA for phosphorylated protein) at 4°C overnight, followed by incubation with Alpaca anti-rabbit IgG-HRP secondary antibody or Goat anti-Mouse IgG-HRP antibody diluted in 3% BSA (1:20000, HUABIO). Images were captured by the e-BLOT Touch Imager (Pro-Life). Densitometric analysis of the protein bands was performed using the ImageJ software.

### BiFC assays

BiFC assays were used for verification of the interaction between eIF2 and GCN2 protein. The whole CDSs of eIF2 and the Conserved Domain Database (CDD) of STKC-eIF2AK4-GCN2 repeat 2 of the serine/threonine kinase of GCN2 (interval 524–906 aa) were amplified by PCR and subcloned into the pie2-YFPC-N1 or pie2-YFPN-C1 vectors [kindly provided by Professor Yutao Xiao (Agricultural Genomics Institute at Shenzhen, Chinese Academy of Agricultural Sciences)] [47], producing YFPN-eIF2 and GCN2-YFPC plasmids, respectively. The specific primers were designed by CE Design (https://crm.vazyme.com/cetool/simple.html) (S5 Table). Sf9 cells was seeded onto a 24-well plate overnight and co-transfected with plasmid pairs (YFPN-eIF2 + GCN2-YFPC, blank plasmid as a control, 500ng each plasmid) using FuGENE HD (Promega) at a ratio of 1:3 (plasmids to FuGENE). Fluorescent signals from the transfected cells were visualized with an inverted confocal microscope (Zeiss LSM980) at 24 h post-transfection.

### Yeast two-hybrid (Y2H) assay

For point-to-point Y2H screen, the bait protein was inserted into the pGBKT7 vector using the In-Fusion Snap Assembly kit (Takara) and successfully expressed in Y2H gold yeast cells without obvious toxicity and self-activation. Similarly, a truncated fragment (524–906 aa) of prey protein GCN2 was cloned into the pGADT7 vector to construct the pGADT7-GCN2 plasmids. Sequence-specific primers are listed in S6 Table. To further confirm the interaction between eIF2 and GCN2, the Y2H gold yeast strain was co-transformed with pGBKT7-eIF2 and pGADT7-GCN2, and serial dilutions of the transformed cells were plated onto DDO (SD/-Leu/-Trp), TDO (SD/-Leu/-Trp/-His) and QDO (SD/-Leu/-Trp/-His/-Ade) media. Y2H gold yeast strains co-transformed with pGBKT7–53 and pGADT7-T, pGBKT7-Lam and pGADT7-T were used as positive and negative controls respectively.

## Supporting information

S1 Raw Data S1 Figure. The Conserved Domain of GCN2. The predicted Conserved Domain marked with black line, RWD–GCN2 (7-126): RWD domain of eIF-2-alpha kinase GCN2 and related proteins. PKC–like super family (250-484): Protein Kinases, catalytic domain. STKC–eIF2AK4–GCN2–rpt2 (524-906): Catalytic domain, repeat 2, of the Serine/Threonine kinase, eukaryotic translation Initiation Factor 2-Alpha Kinase 4 or General Control Non-derepressible-2. Class–II–aaRS-like-core sup er family (968-1294): Class II tRNA amino-acyl synthetase-like catalytic core domain. (XLSX)

S1 Fig The Conserved Domain of GCN2. The predicted Conserved Domain marked with black line, RWD–GCN2 (7–126): RWD domain of eIF-2-alpha kinase GCN2 and related proteins. PKC–like super family (250–484): Protein Kinases, catalytic domain. STKC–eIF2AK4–GCN2–rpt2 (524–906): Catalytic domain, repeat 2, of the Serine/Threonine kinase, eukaryotic translation Initiation Factor 2-Alpha Kinase 4 or General Control Non-derepressible-2. Class–II–aaRS-like-core sup er family (968–1294): Class II tRNA amino-acyl synthetase-like catalytic core domain.
(DOCX)

S1 Table Primer sequences used for cloning. (DOCX)

S2 Table The accession numbers of GCN2 genes used in the neighbor-joining tree analysis. (DOCX)

S3 Table Primer sequences used for qPCR. (DOCX)

S4 Table Primer sequences used for dsRNA templates. Underlined T7 promoter sequences.
(DOCX)

S5 Table Primer sequences used for recombinant BiFc plasmids. Small letters indicate homologous arm sequence of pie2-YFPC-N1 or pie2-YFPN-C1 vectors.
(DOCX)

S6 Table Primer sequences used for recombinant Y2H plasmids. Underlined homologous arm sequence of pGBKT7 and pGADT7 vectors.
(DOCX)
