# Supplementary material for: The interaction between GCN2 and eIF2 mediates the resistance of cotton bollworm to the Bacillus thuringiensis Cry1Ac toxin
Source: PLoS Pathog. 2025 Sep 15;21(9):e1013510. doi: 10.1371/journal.ppat.1013510 (PMC12448995; doi:10.1371/journal.ppat.1013510)

**S1 Fig. The Conserved Domain of GCN2.** The predicted Conserved Domain marked with black line, RWD–GCN2 (7-126): RWD domain of eIF-2-alpha kinase GCN2 and related proteins. PKC–like super family (250-484): Protein Kinases, catalytic domain. STKC–eIF2AK4–GCN2–rpt2 (524-906): Catalytic domain, repeat 2, of the Serine/Threonine kinase, eukaryotic translation Initiation Factor 2-Alpha Kinase 4 or General Control Non-derepressible-2. Class–II–aaRS-like-core sup er family (968-1294): Class II tRNA amino-acyl synthetase-like catalytic core domain.


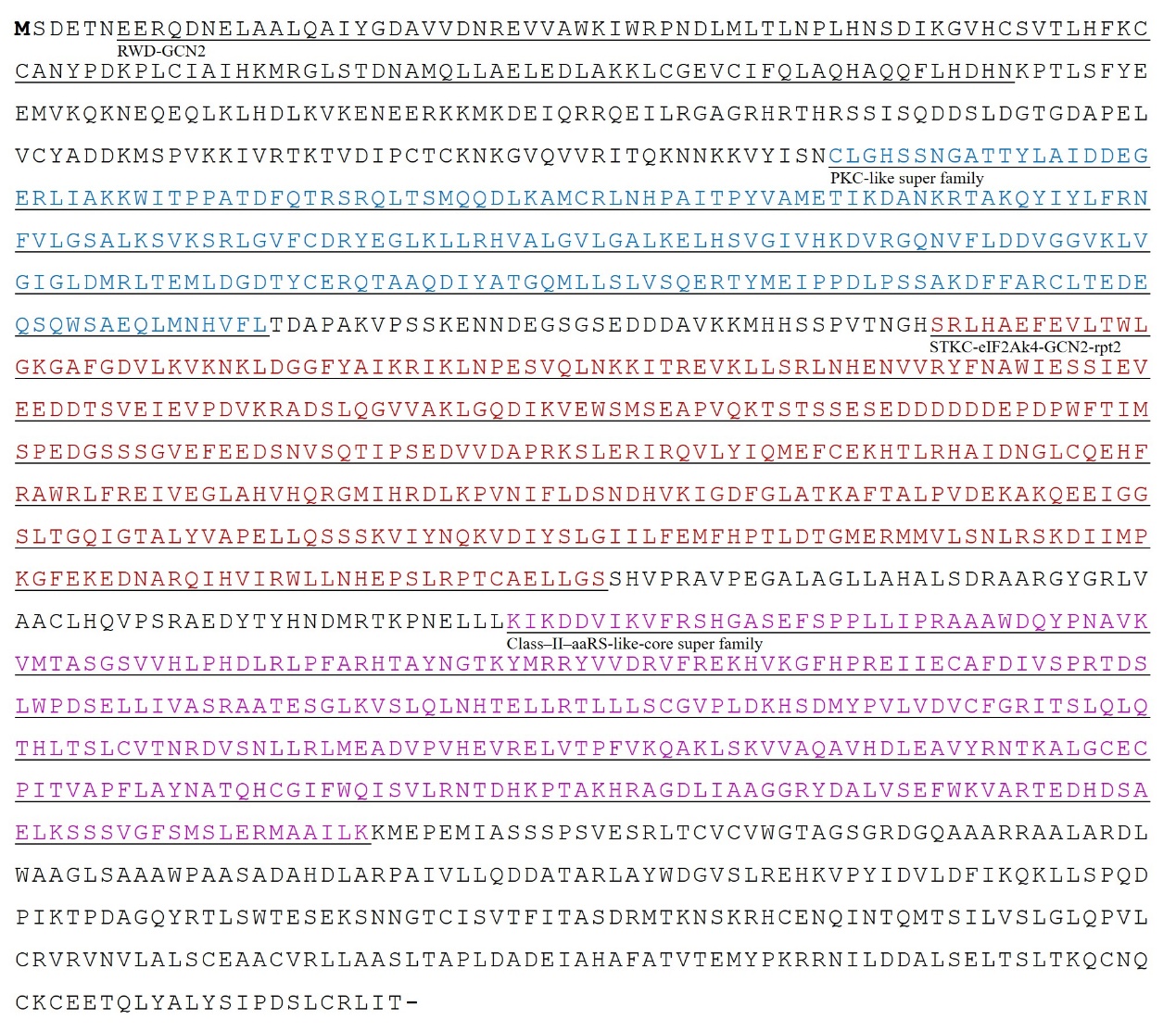

Supplement: S1 Fig — The predicted Conserved Domain marked with black line, RWD–GCN2 (7–126): RWD domain of eIF-2-alpha kinase GCN2 and related proteins. PKC–like super family (250–484): Protein Kinases, catalytic domain. STKC–eIF2AK4–GCN2–rpt2 (524–906): Catalytic domain, repeat 2, of the Serine/Threonine kinase, eukaryotic translation Initiation Factor 2-Alpha Kinase 4 or General Control Non-derepressible-2. Class–II–aaRS-like-core sup er family (968–1294): Class II tRNA amino-acyl synthetase-like catalytic core domain. (DOCX) [file ppat.1013510.s002.docx]
