# Supplementary material for: The interaction between GCN2 and eIF2 mediates the resistance of cotton bollworm to the Bacillus thuringiensis Cry1Ac toxin
Source: PLoS Pathog. 2025 Sep 15;21(9):e1013510. doi: 10.1371/journal.ppat.1013510 (PMC12448995; doi:10.1371/journal.ppat.1013510)
Supplement: S1 Table — (DOCX) [file ppat.1013510.s003.docx]

**S1 Table. Primer sequences used for cloning**

| GCN2-F | ATGAGTGACGAAACGAACGA |
| --- | --- |
| GCN2-R | TCAAGTGATCAACCGGCATAGA |
