# Supplementary material for: The interaction between GCN2 and eIF2 mediates the resistance of cotton bollworm to the Bacillus thuringiensis Cry1Ac toxin
Source: PLoS Pathog. 2025 Sep 15;21(9):e1013510. doi: 10.1371/journal.ppat.1013510 (PMC12448995; doi:10.1371/journal.ppat.1013510)
Supplement: S2 Table — (DOCX) [file ppat.1013510.s004.docx]

**S2 Table. The accession numbers of GCN2 genes used in the neighbor-joining tree analysis.**

| Genes | Species | Accession number |
| --- | --- | --- |
| HaGCN2 | *Helicoverpa armigera* | XP_049704467.1 |
| HzGCN2 | *Helicoverpa zea* | XP_047034176.1 |
| SfGCN2 | *Spodoptera frugiperda* | XP_050551368.1 |
| SlGCN2 | *Spodoptera litura* | XP_022835131.1 |
| TnGCN2 | *Trichoplusia ni* | XP_026743008.1 |
| OfGCN2 | *Ostrinia furnacalis* | XP_028168706.1 |
| PgGCN2 | *Pectinophora gossypiella* | XP_049878089.1 |
| BmGCN2 | *Bombyx mori* | XP_004923430.1 |
| HkGCN2 | *Hyposmocoma kahamanoa* | XP_026333325.1 |
| PiGCN2 | *Plodia interpunctella* | XP_053613620.1 |
| ZcGCN2 | *Zerene cesonia* | XP_038206892.1 |
| CcGCN2 | *Colias croceus* | XP_045502647.1 |
| PnGCN2 | *Pieris napi* | XP_047512690.1 |
| PrGCN2 | *Pieris rapae* | XP_045486079.1 |
| PbGCN2 | *Pieris brassicae* | XP_045533052.1 |
| NiGCN2 | *Nymphalis io* | XP_050354452.1 |
| MsGCN2 | *Manduca sexta* | XP_037294146.1 |
| LsGCN2 | *Leptidea sinapis* | XP_050664185.1 |
| AtGCN2  MjGCN2  MhGCN2  VaGCN2  VtGCN2  VcGCN2  AaGCN2  AgGCN2  PaGCN2  BaGCN2  DpGCN2  GmGCN2  McGCN2  EjGCN2  PxGCN2  CsGCN2  VpGCN2  VvGCN2  VmGCN2  NlGCN2  VveGCN2  VcrGCN2  NfGCN2  NpGCN2  DsGCN2  NvGCN2  CciGCN2  SpGCN2  PcGCN2  SsGCN2  DnGCN2 | *Amyelois transitella*  *Maniola jurtina*  *Maniola hyperantus*  *Vanessa atalanta*  *Vanessa tameamea*  *Vanessa cardui*  *Aricia agestis*  *Achroia grisella*  *Pararge aegeria*  *Bicyclus anynana*  *Danaus plexippus*  *Galleria mellonella*  *Melitaea cinxia*  *Eumeta japonica*  *Plutella xylostella*  *Cryptotermes secundus*  *Vespula pensylvanica*  *Vespula vulgaris*  *Vespa mandarinia*  *Neodiprion lecontei*  *Vespa velutina*  *Vespa crabro*  *Neodiprion fabricii*  *Neodiprion pinetum*  *Diprion similis*  *Neodiprion virginianus*  *Cephus cinctus*  *Schistocerca piceifrons*  *Polistes Canadensis*  *Schistocerca serialis*  *Drosophila nasuta* | XP_060805089.1  XP_045776923.1  XP_034834138.1  XP_047536765.1  XP_026494223.1  XP_046971420.1  XP_041969530.1  XP_059048217.1  XP_039757544.1  XP_023949864.2  XP_032521153.1  XP_052756419.1  XP_045453243.1  GBP22173.1  XP_048486335.1  XP_023718264.1  XP_043673456.1  XP_050857131.1  XP_035732758.1  XP_046601274.1  XP_047357227.1 XP_046825418.1  XP_046434813.1  XP_046490900.1  XP_046750466.1  XP_046628055.1  XP_015584813.1  XP_047111847.1  XP_014616542.1  XP_049955903.1  XP_060650693.1 |
