# Supplementary material for: The interaction between GCN2 and eIF2 mediates the resistance of cotton bollworm to the Bacillus thuringiensis Cry1Ac toxin
Source: PLoS Pathog. 2025 Sep 15;21(9):e1013510. doi: 10.1371/journal.ppat.1013510 (PMC12448995; doi:10.1371/journal.ppat.1013510)
Supplement: S3 Table — (DOCX) [file ppat.1013510.s005.docx]

**S3 Table. Primer sequences used for qPCR.**

| qGCN2-F | CGCTCTAGCCCGGGATCT | Amplification efficiency (%)  101.2 |
| --- | --- | --- |
| qGCN2-R | GCAGTAGCATCGTCTTGCAG |  |
| RPS15-F | CTGAGGTCGATGAAACTCTC | 100 |
| RPS15-R | CTCCATGAGTTGCTCATTG |  |
