# Supplementary material for: The interaction between GCN2 and eIF2 mediates the resistance of cotton bollworm to the Bacillus thuringiensis Cry1Ac toxin
Source: PLoS Pathog. 2025 Sep 15;21(9):e1013510. doi: 10.1371/journal.ppat.1013510 (PMC12448995; doi:10.1371/journal.ppat.1013510)
Supplement: S4 Table — Underlined T7 promoter sequences. (DOCX) [file ppat.1013510.s006.docx]

**S4 Table. Primer sequences used for dsRNA templates.** Underlined T7 promoter sequences.

| dsGCN2-F | GATCACTAATACGACTCACTATAGGGAGACCATACCTTCGGAGGACGTA |
| --- | --- |
| dsGCN2-R | GATCACTAATACGACTCACTATAGGGAGAATCCTTTCCATCCCCGTATC |
