# Supplementary material for: The interaction between GCN2 and eIF2 mediates the resistance of cotton bollworm to the Bacillus thuringiensis Cry1Ac toxin
Source: PLoS Pathog. 2025 Sep 15;21(9):e1013510. doi: 10.1371/journal.ppat.1013510 (PMC12448995; doi:10.1371/journal.ppat.1013510)
Supplement: S5 Table — Small letters indicate homologous arm sequence of pie2-YFPC-N1 or pie2-YFPN-C1 vectors. (DOCX) [file ppat.1013510.s007.docx]

**S5 Table. Primer sequences used for recombinant BiFc plasmids.** Small letters indicate homologous arm sequence of pie2-YFPC-N1 or pie2-YFPN-C1 vectors.

| YFPC-N1/C1-F | GCTAAACGTATTAAGAAGTCCAGTTCA |
| --- | --- |
| YFPC-N1/C1-R  BiFc- GCN2-F  BiFc- GCN2-R | AAGAGCTCGGTACCTATCGATAGAG  tcgataggtaccgagctcttATGTCACGTCTGCACGCTGAG  gacttcttaatacgtttagcACTGCCTAGAAGTTCGGCG |
