# Supplementary material for: The interaction between GCN2 and eIF2 mediates the resistance of cotton bollworm to the Bacillus thuringiensis Cry1Ac toxin
Source: PLoS Pathog. 2025 Sep 15;21(9):e1013510. doi: 10.1371/journal.ppat.1013510 (PMC12448995; doi:10.1371/journal.ppat.1013510)
Supplement: S6 Table — Underlined homologous arm sequence of pGBKT7 and pGADT7 vectors. (DOCX) [file ppat.1013510.s008.docx]

**S6 Table. Primer sequences used for recombinant Y2H plasmids.** Underlined homologous arm sequence of pGBKT7 and pGADT7 vectors.

| pGBKT7- eIF2-F | TGATCTCAGAGGAGGACCTGCATATGCCTCTTTCGTGTCGATTTTACCAAGAAAAG |
| --- | --- |
| pGBKT7-eIF2-R  pGADT7-GCN2-F  pGADT7-GCN2-R | TGCGGCCGCTGCAGGTCGACGGATCCCTAATCCTCGTCTTCATCGGACGCTC  ACGACGTACCAGATTACGCTCATATGTCACGTCTGCACGCTGAGTTTGAAGTCCTCACG  GGATCCCGTATCGATGCCCACCCGGGTCAACTGCCTAGAAGTTCGGCGCAAGTTGGACG |
